# Plasminogen binding and activation at the breast cancer cell surface: the integral role of urokinase activity

**DOI:** 10.1186/bcr1647

**Published:** 2007-01-28

**Authors:** Gillian E Stillfried, Darren N Saunders, Marie Ranson

**Affiliations:** 1 School of Biological Sciences, University of Wollongong, Northfields Avenue, Wollongong, NSW, 2522, Australia; 2 Cancer Research Program, Garvan Institute of Medical Research, 384 Victoria Street, Darlinghurst, NSW, 2010, Australia

## Abstract

**Introduction:**

The regulation of extracellular proteolytic activity via the plasminogen activation system is complex, involving numerous activators, inhibitors, and receptors. Previous studies on monocytic and colon cell lines suggest that plasmin pre-treatment can increase plasminogen binding, allowing the active enzyme to generate binding sites for its precursor. Other studies have shown the importance of pre-formed receptors such as annexin II heterotetramer. However, few studies have used techniques that exclusively characterise cell-surface events and these mechanisms have not been investigated at the breast cancer cell surface.

**Methods:**

We have studied plasminogen binding to MCF-7 in which urokinase plasminogen activator receptor (uPAR) levels were upregulated by PMA (12-*O*-tetradecanoylphorbol-13-acetate) stimulation, allowing flexible and transient modulation of cell-surface uPA. Similar experiments were also performed using MDA-MB-231 cells, which overexpress uPAR/uPA endogenously. Using techniques that preserve cell integrity, we characterise the role of uPA as both a plasminogen receptor and activator and quantify the relative contribution of pre-formed and cryptic plasminogen receptors to plasminogen binding.

**Results:**

Cell-surface plasminogen binding was significantly enhanced in the presence of elevated levels of uPA in an activity-dependent manner and was greatly attenuated in the presence of the plasmin inhibitor aprotinin. Pre-formed receptors were also found to contribute to increased plasminogen binding after PMA stimulation and to co-localise with uPA/uPAR and plasminogen. Nevertheless, a relatively modest increase in plasminogen-binding capacity coupled with an increase in uPA led to a dramatic increase in the proteolytic capacity of these cells.

**Conclusion:**

We show that the majority of lysine-dependent plasminogen binding to breast cancer cells is ultimately regulated by plasmin activity and is dependent on the presence of significant levels of active uPA. The existence of a proteolytic positive feedback loop in plasminogen activation has profound implications for the ability of breast cancer cells expressing high amounts of uPA to accumulate a large proteolytic capacity at the cell surface, thereby conferring invasive potential.

## Introduction

The components of the plasminogen activation system (PAS) are important determinants of metastatic capacity, participating in both proteolytic and non-proteolytic pathways during cancer progression [1,2]. Plasminogen (plg), the central zymogen in the PAS, is secreted as a single-chain glycosylated protein with an N-terminal glutamic acid (Glu) residue, five kringle regions containing lysine-binding sites that regulate plg binding and activation (kringles 1, 4, and 5), and a C-terminal protease domain [3]. Plg may be activated to the broad-spectrum protease plasmin (pln) by a number of proteases, including tissue-type plg activator (tPA), factor XIa, factor XIIa, and kallikrein, via cleavage of the Arg_561_-Val_562 _peptide bond [4]. However, the urokinase plg activator (uPA) is widely accepted as the most significant activator of plg during tissue degradation [5,6]. This serine protease is secreted as the 53-kDa zymogen pro-uPA and is present at the cell surface bound to its GPI (glycosylphosphatidylinositol)-anchored receptor, uPAR [5]. Receptor-bound pro-uPA is activated by pln and a number of other proteases *in vitro *through cleavage of the Lys_158_-Ile_159 _peptide bond to form the active two-chain protease uPA. The uPA A-chain contains a growth factor-like domain (amino acids 1 to 48) and a kringle domain (amino acids 50 to 131), whereas the B-chain contains the protease domain [7]. The reciprocal activation of pro-uPA by pln and of plg by uPA is an important mechanism in the regulation of pln activity [8].

Receptor-mediated cell-surface localisation of the various components of the PAS (for example, uPA, plg, and plg activator inhibitor type 1 [PAI-1] and type 2 [PAI-2]) is critical for the spatial and temporal regulation of proteolysis. Proteins, gangliosides, and free fatty acids are among the mediators that regulate cell-surface plg binding [9,10]. Several heterogeneous candidate receptors have been identified, including actin [11], amphoterin [12], annexin II heterotetramer (AIIt) [13], cytokeratin 8/18 [14], and α-enolase [15,16], with dissociation constant (*K*_d_) values ranging from 0.1 to 2 μM. Receptor candidates can be grouped into three classes: (a) those that possess a pre-existing C-terminal lysine residue (pre-formed), (b) those that are cleaved to expose a lysine residue (cryptic), and (c) those that bind plg through a lysine-independent mechanism [1]. Initial binding of Glu-plg to exposed internal or C-terminal lysine residues results in a rapid conformational change, whereas binding at a second lysine residue stabilises the more open, activation-susceptible γ-conformation [17-19]. In addition, treatment of cells with basic caboxypeptidases significantly reduces plg binding [20]. The lysine-dependent binding of plg via cell-surface receptors therefore both anchors plg to the cell surface and facilitates its activation to pln. The high plg-binding capacity (10^4 ^to 10^7 ^binding sites per cell) and relatively low affinity (*K*_d _0.1 to 2 μM) of cell-surface plg binding suggest the presence of multiple plg receptors that are responsible for the total plg-binding capacity of a cell [1]. Furthermore, several studies have shown that limited proteolysis of the cell surface with trypsin or pln to reveal C-terminal lysyl residues results in enhanced plg binding in various cell types [21-23]. Together, these observations point to a general mechanism of cell-surface plg binding in which total binding is determined by the collective expression of a spectrum of heterogeneous receptors and proteolytic activity.

Mounting evidence implicates uPA as a key regulator of breast cancer metastasis and demonstrates a role for uPA in a number of processes that facilitate tumour progression, including extracellular matrix degradation, cell proliferation, migration, and adhesion [24,25]. Furthermore, high levels of uPA and its inhibitor PAI-1 in primary breast tumours are correlated with shortened disease-free interval and poor overall survival, independent of other predictors such as tumour grade, tumour size, and hormone receptor status [2,26]. Significantly higher levels of uPA and uPAR are expressed at the cell surface of metastatic breast cancer cells *in vitro *and these cells are capable of binding larger amounts of Glu-plg at the cell surface than non-metastatic breast cancer cells [27]. Moreover, although plg displays widespread cell-surface binding to a range of putative receptors, it is the uPA-co-localised fraction of plg that is readily activated to pln [28]. This highlights uPA as an important regulator of cellular plg activation.

A direct, non-active-site interaction between the uPA A-chain and plg has been shown to exist at the cell surface of the monocytic cell line, U937 [29]. In addition, the amino-terminal fragment of uPA (specifically the Lys_135 _residue) has been shown to mediate the activation of plg by uPA and the reciprocal activation of pro-uPA by pln [30]. Thus, the accrual of active uPA may be a key step in tumour invasion and metastasis by controlling not only the levels of plg activated at the cell surface but also the levels of plg bound. This mechanism has not been definitively shown *in situ *(that is, at the cell surface) on breast cancer cells. Using techniques that preserve cell integrity and exclude the contribution of intracellular plg-binding moieties [28], we characterise the relative contribution of uPA, pln, and several candidate plg receptors to cell-surface plg binding on breast cancer cells.

## Materials and methods

### Specific proteins and antibodies

Active human urine-derived uPA (40% high molecular weight, 50% low molecular weight, 10% transitory forms; A-chain C-terminal residue: Phe_158_) and mouse anti-cytokeratin 8/18 monoclonal antibody (mAb) (CBL195) were purchased from Chemicon International (Temecula, CA, USA). Bovine aprotinin, rabbit anti-actin C-11 polyclonal antibody (pAb) (A2066), rabbit immunoglobulin G (IgG) (I5006), goat IgG (I9140), fluorescein isothiocyanate (FITC)-labelled goat anti-mouse F_ab_-specific pAb (F4018), FITC-labelled rabbit anti-goat pAb (F7367), FITC-labelled goat anti-rabbit pAb (F6005), and cyanine (Cy) 3-labelled goat anti-mouse pAb (C2181) were purchased from Sigma-Aldrich Pty. Ltd. (St. Louis, MO, USA). Spectrozyme PL, mouse anti-uPAR mAb (no. 3936), mouse anti-uPA A-chain mAb (no. 3921), and mouse anti-uPA B-chain mAb (no. 394) were purchased from American Diagnostica Inc. (Stamford, CT, USA). Goat anti-S100A10 (p11) mAb (AF1698) was purchased from R&D Systems, Inc. (Minneapolis, MN, USA). Human α_2_-antiplasmin was purchased from Molecular Innovations, Inc. (Southfield, MI, USA). Goat anti-annexin II (C-16) pAb (sc-1924) was purchased from Santa Cruz Biotechnology, Inc. (Santa Cruz, CA, USA). Irrelevant isotype-matched control antibodies mouse anti-sheep lymphocyte antigen IgG_1 _mAb (SBU-T6) and IgG_2α _mAb (SBU-LCA) were obtained from the Centre for Animal Biotechnology (Parkville, Victoria, Australia).

### Cell culture

MCF-7 (low uPAR/uPA-expressing, low plg-binding) and MDA-MB-231 (high uPAR/uPA-expressing, high plg-binding) cell lines were maintained in RPMI-1640 medium (Invitrogen Corporation, Carlsbad, CA, USA) supplemented with 5% heat-inactivated foetal calf serum (FCS) (Thermo Electron Corporation, Waltham, MA, USA) at 37°C in a humidified incubator with 5% CO_2_.

### Purification and modification of Glu-plg

Human Glu-plg was affinity-purified from plasma using Lysine Sepharose™ 4B (GE Healthcare, Little Chalfont, Buckinghamshire, UK) as described [16]. Glu-plg was either FITC- or Cy5-conjugated as described [28,31].

### Inactivation of uPA

Purified human uPA (0.5 mg/ml) was inactivated by incubation overnight with alpha-toluenesulfonyl fluoride (PMSF) (1 mM) in phosphate-buffered saline (PBS) (pH 7.4) at 4°C (final concentration of isopropanol 2%). PMSF inactivation of uPA was maintained for the duration of the assays (data not shown). Pln activity assays confirmed that residual excess PMSF in the preparation was negligible after overnight incubation and dilution for use in the assays (data not shown).

### PMA treatment to enhance cell-surface uPA

MCF-7 cells were cultured for 32 hours in 5% FCS/RPMI-1640 and then incubated with PMA (12-*O*-tetradecanoylphorbol-13-acetate) (100 nM; concentration of ethanol 0.006% vol/vol) without change of media at 37°C for a further 16 hours as previously described [32]. Control cells were treated with an equivalent volume of ethanol in 5% FCS/RPMI-1640. Cells were characterised for plg binding and activation and PAS component expression as described below. Ethanol has been shown to increase uPAR expression in certain cell types [33], but this effect was not observed in MCF-7 at the low concentration used (data not shown).

### Cell-surface plg binding assays

Throughout this study, only the lysine-dependent proportion of total plg binding is shown because this represents the pool of cell-bound plg which is readily activatable [28]. Subconfluent, adherent cells were prepared and analysed for lysine-dependent plg binding essentially as described [27]. However, prior to incubation with antibodies or plg, cells were pre-incubated with either uPA or PMSF-inactivated uPA (PMSF-uPA) (50 nM) in binding buffer (Hanks' balanced salt solution containing 1 mM calcium chloride, 1 mM magnesium chloride, 20 mM HEPES [N-(2-hydroxyethyl)piperazine-N'-(2-ethanesulfonic acid)], and 0.1% bovine serum albumin) (pH 7.4) for 10 minutes at room temperature (21°C to 25°C) (allowing limited proteolytic activity) and then washed prior to further analysis on ice with all incubations performed in the absence or presence of aprotinin (1.6 trypsin inhibitor units [TIU]). Lysine-dependent plg binding was calculated as total plg binding minus binding in the presence of 1 mM tranexamic acid. Lysine-dependent binding was typically 58% ± 3% of total cell-surface binding in the MCF-7 cell line and 83% ± 1% in the MDA-MB-231 cell line. For antibody inhibition experiments, cells were pre-incubated for 30 minutes with 20 μg/ml anti-uPA A-chain (mAb no. 3921), anti-uPA B-chain (mAb no. 394), or an irrelevant isotype control on ice prior to the plg binding step. Cells were then washed and analysed for cell-surface lysine-dependent plg binding as described [27].

Plg binding assays were performed using dual-colour flow cytometry with propidium iodide (PI) to distinguish between viable and non-viable cells as previously described [27]. Only cells that excluded PI (that is, viable cells with intact membranes) were included in further analyses. Cell-surface-associated fluorescence was measured on an LSR II flow cytometer (BD Biosciences, San Jose, CA, USA). All data were analysed using WinMDI version 2.8 (The Scripps Research Institute, La Jolla, CA, USA) [34].

### Plg activation assays

MCF-7 cells were grown, PMA-treated, and pre-incubated with uPA or PMSF-uPA (50 nM) for 10 minutes at room temperature as per plg binding assays in modified (no phenol red) binding buffer (MBB) (pH 7.4). Cells were then washed twice and resuspended in MBB containing 0.5 μM Glu-plg in the presence and absence of 1.6 TIU aprotinin (to measure pln-dependent effects). Cells were incubated for 10 minutes at room temperature before addition of 0.4 μM of the colorimetric pln substrate Spectrozyme PL. Plg activation assays were performed in the presence of 0.25 μM α_2_-antiplasmin to inhibit pln activity in solution and to ensure that only cell-surface pln activity was assayed. Pln activity was measured at 405 nm at 37°C using a Spectramax 250 plate reader with Softmax Pro version 4.0 software (Molecular Devices Corporation, Sunnyvale, CA, USA).

### Cell-surface pre-formed receptor characterisation by flow cytometry and confocal microscopy

Cell-surface uPAR, uPA, cytokeratin 8/18, actin, annexin II, and S100A10 (p11) expression was analysed by indirect immunofluorescence staining as previously described [27]. Only cells that excluded PI (that is, viable cells with intact membranes) were included in further analyses. Isotype control fluorescence was subtracted from all measurements. These antibodies have previously been shown to detect their respective antigens on positive controls [28].

For confocal microscopy, MCF7 cells (2 × 10^5 ^cells per millilitre) were cultured onto sterile glass coverslips in 24-well tissue culture plates in 1 ml of 5% FCS/RPMI-1640 for 32 hours and PMA treated as above. Coverslips were washed twice with ice-cold MBB and fixed by incubation with freshly prepared, ice-cold 3.75% paraformaldehyde in PBS (pH 7.4) for 15 minutes on ice. Coverslips were washed twice and blocked for 30 minutes with MBB on ice. Samples were incubated for 10 minutes at room temperature with MBB containing 50 nM uPA. Cells were washed and incubated for 45 minutes with Cy5-labelled Glu-plg (0.5 μM) in the presence and absence of 5 mM tranexamic acid in MBB on ice in the absence of light. Cells were washed and incubated for 30 minutes with 10 to 20 μg/ml mouse anti-uPAR mAb (no. 3936), mouse anti-uPA mAb (no. 394), or goat anti-annexin II (C-16) pAb (sc-1924) in MBB on ice in the absence of light. After two washes, samples were incubated for 30 minutes either with 1:50 FITC- or Cy3-labelled goat anti-mouse pAb or with FITC-labelled rabbit anti-goat pAb in MBB on ice in the absence of light and washed three times with ice-cold MBB. Samples were mounted and examined as previously described [28] using a × 40/1.00 FLUOTAR PL oil immersion objective lens on a Leica DM IRBE confocal microscope (Leica Microsystems GmbH, Wetzlar, Germany). All images were analysed using Confocal Assistant version 4.02 (Todd Clark Brelje, University of Minnesota, Minneapolis, MN, USA).

### Statistics

Graphs shown represent mean ± standard error (performed in duplicate) and are representative of experiments performed on multiple days. The means of the data were compared statistically using Student's *t *test (assuming equal variances). Where variances differed significantly, data were log-transformed prior to analysis by *t *test.

## Results

### Plg binds directly to endogenous (pro)-uPA

**Figure 1:**
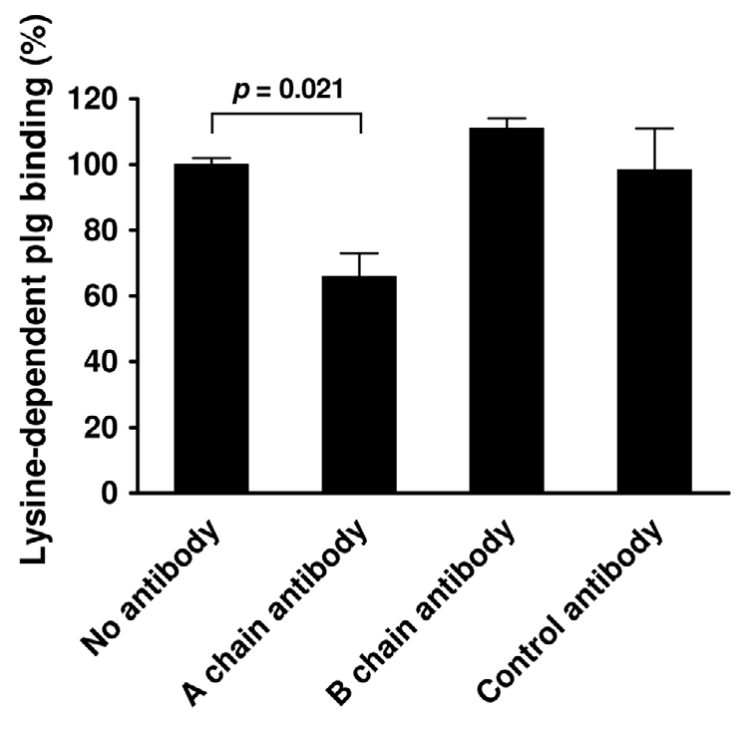
Cell-surface plasminogen (plg) binding via (pro)-uPA on MDA-MB-231 cells. MDA-MB-231 cells were detached, washed, and pre-incubated on ice for 30 minutes with antibodies against the uPA A-chain, the uPA B-chain, or an irrelevant isotype control antibody. Cells were then washed and incubated for 45 minutes on ice in the dark with fluorescein isothiocyanate-labelled Glu-plg in the absence (total binding) and presence (lysine-independent binding) of 1 mM tranexamic acid. Cells were then washed and analysed for cell-surface plg binding in the presence of 5 μg/ml of the vital stain, propidium iodide, using dual-colour flow cytometry. Lysine-dependent plg binding was calculated by subtracting lysine-independent binding from total binding. Glu, glutamic acid; uPA, urokinase plasminogen activator.

The presence of a non-active site, direct binding determinant for plg in the C-terminal region (Gly_149_-Lys_158_) of the uPA A-chain has been shown to exist on U937, a monocytic cell line [29], and we have previously shown that uPA and plg co-localise at the surface of breast cancer cells [28]. To determine whether this interaction occurs at the surface of MDA-MB-231 cells (a breast cancer cell line that has high endogenous uPA expression [27]), binding experiments using antibodies directed against the A- or B-chain of uPA were performed under conditions that predominantly exclude proteolytic activation of plg by uPA. A significant decrease (34%) in cell-surface lysine-dependent plg binding was observed only in the presence of anti-uPA A-chain mAb (Figure 1), indicating the presence of a plg-binding site within this domain of uPA. To confirm that the A-chain uPA antibody used did not dissociate bound uPA, thus leading to the observed decrease in plg binding, we performed these blocking experiments using stimulated MCF-7 cells with significant levels of cell-surface urine-derived uPA, which lacks the C-terminal lysine (see below). Under these conditions, plg binding was not decreased (data not shown), which also confirms the importance of the C-terminal lysine for binding. Interestingly, under similar conditions, uPAR-transfected T-47D cells [35], which display concomitant but small and variable increases in endogenous uPA (1.7 ± 0.23-fold), also displayed small but variable increases in plg-binding capacity (1.7 ± 0.19-fold).

### Modulation of cell-surface uPA on MCF-7 cells

**Figure 2:**
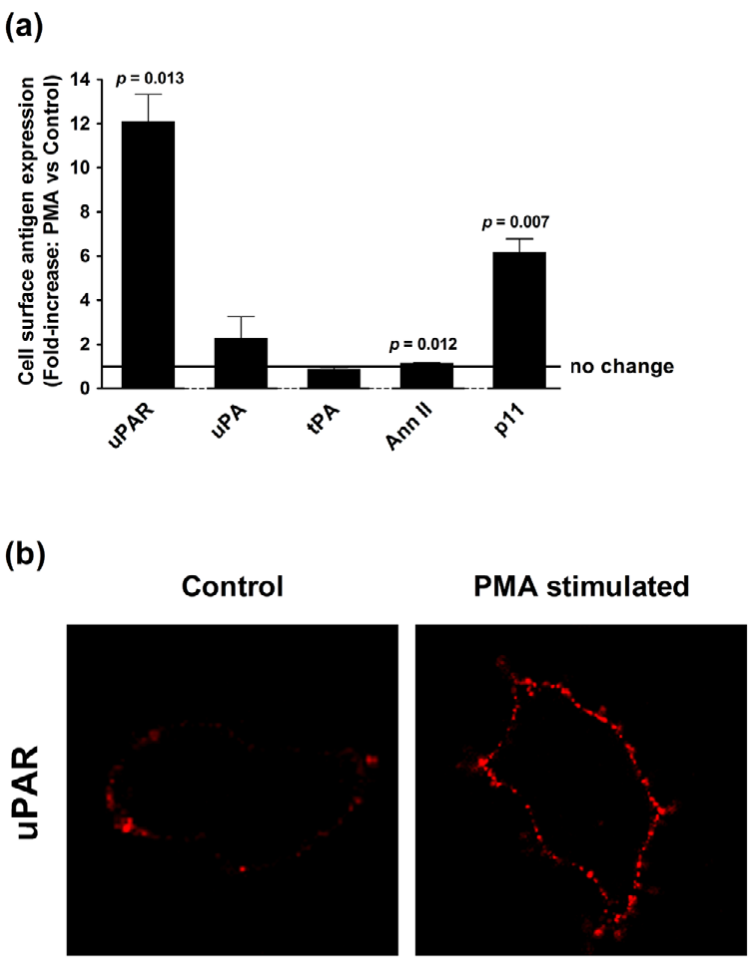
Plasminogen activation system component changes on PMA-treated MCF-7 cells. MCF-7 cells were cultured for 16 hours in 5% foetal calf serum/RPMI containing 100 nM PMA. **(a) **Cells were washed and incubated for 30 minutes on ice with 10 to 20 μg/ml of anti-uPAR monoclonal antibody (mAb), anti-uPA mAb, anti-tPA mAb, anti-annexin II (Ann II) C-16 polyclonal antibody (pAb), anti-S100A10 (p11) pAb, or a matched isotype control antibody. Cells were then washed and incubated for 45 minutes on ice in the dark with a 1:200 dilution of appropriate secondary antibody labelled with fluorescein isothiocyanate (FITC). All cells were then washed and analysed for cell-surface-associated FITC fluorescence in the presence of 5 μg/ml propidium iodide using dual-colour flow cytometry. Specific antibody binding was calculated by subtracting control antibody fluorescence. Solid line indicates a fold increase of 1 (equivalent to no change). *P *values indicate a significant increase compared to the corresponding untreated control. **(b) **Confocal microscopy on attached cells was also used to verify enhancement of cell-surface uPAR by PMA treatment. PMA, 12-O-tetradecanoylphorbol 13-acetate; tPA, tissue-type plasminogen activator; uPA, urokinase plasminogen activator; uPAR, urokinase plasminogen activator receptor.

High levels of uPA on breast cancer cell lines are linked to increased plg binding [27,28]. We and others [36] have found that acid stripping of the cell surface to remove uPA markedly compromises membrane integrity, making cell-surface measurements difficult and increasing the likelihood of introducing artifacts. In addition, upregulation of uPAR using transfection techniques [35] did not allow sufficient control of uPA levels between experiments (data not shown). Therefore, to directly assess the effect of altered cell-surface uPA on plg-binding capacity, we used a model breast cancer cell line (MCF-7) that has very low constitutive cell-surface uPAR and modulated its expression by PMA stimulation [32]. After 16 hours of stimulation with 100 nM PMA, a large enhancement (approximately 12-fold; mean fluorescence intensity [MFI] of 3.4 ± 1.2 versus 41.4 ± 4.2 fluorescence units after PMA stimulation) of cell-surface uPAR was observed on MCF-7 cells (Figure 2a,b). Enhancement of uPAR expression was observed without significant changes to endogenous cell-surface expression of tPA (Figure 2a), actin, or cytokeratin 8/18 (data not shown), which had low to non-detectable expression. Neither PAI-1 nor PAI-2 was detectable at significant levels at the cell surface under any condition (data not shown). Increases in endogenous uPA after PMA stimulation were small (twofold; MFI of 6.19 ± 2.07 versus 14.2 ± 6.0 fluorescence units after PMA stimulation) but not statistically significant. Although the increase in annexin II (1.4-fold) was also relatively small, this observation is significant because basal annexin II expression was high (MFI of 782 ± 5 versus 895 ± 17 after PMA stimulation). Interestingly, a substantial (6.1-fold) increase in S100A10 (p11) expression was also observed after PMA stimulation (Figure 2a). However, it should be noted that overall expression levels of p11 were still low (MFI of 1.74 ± 0.26 versus 10.73 ± 1.04 fluorescence units after PMA stimulation).

**Figure 3:**
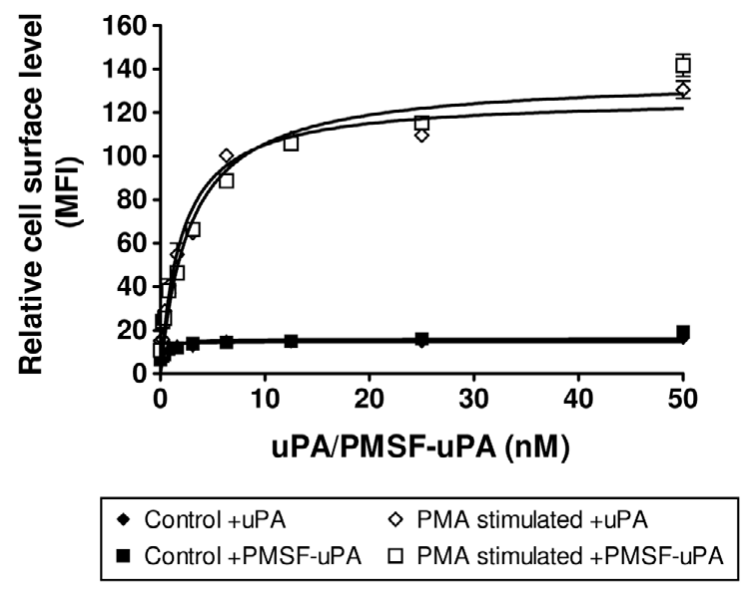
Cell-surface urokinase plasminogen activator (uPA) binding on PMA-stimulated cells. MCF-7 cells were cultured for 16 hours in 5% foetal calf serum/RPMI containing 100 nM PMA (open symbols) or vehicle control (closed symbols). Cells were detached, washed, and pre-incubated for 30 minutes on ice with increasing concentrations of active uPA or PMSF-inactivated uPA. Cells were then washed and analysed for cell-surface uPA level as in Figure 2. Curves were fitted using a one-site binding equation in GraphPad Prism version 4.00 (GraphPad Software, Inc., San Diego, CA, USA). MFI, mean fluorescence intensity; PMA, 12-O-tetradecanoylphorbol 13-acetate; PMSF, alpha-toluenesulfonyl fluoride.

Dose-dependent cell-surface binding of exogenous uPA was observed on PMA-stimulated MCF-7 cells. Saturation of enhanced uPAR was observed at 50 nM uPA (Figure 3), representing an approximately eightfold increase in cell-surface uPA compared to untreated controls. PMSF-uPA displayed identical cell-surface binding properties to active uPA (Figure 3), indicating that inactivation did not affect the binding characteristics of uPA. Several studies have suggested that uPA may cleave cell-surface uPAR, rendering it incapable of binding uPA and its other binding partners [37-39]. However, we observed no change in the level of cell-surface uPAR subsequent to incubation with uPA (data not shown). Thus, stimulation of MCF-7 cells with PMA for 16 hours followed by incubation with 50 nM uPA provides appropriate flexibility in transient modulation of cell-surface uPA. Furthermore, the use of urine-derived uPA eliminates significant A-chain Lys_158 _residue contribution to plg binding and thus this model is highly useful for investigating the role of uPA activity in cell-surface plg binding and activation.

### Spatial organisation of cell-surface plg binding and putative plg receptors

**Figure 4:**
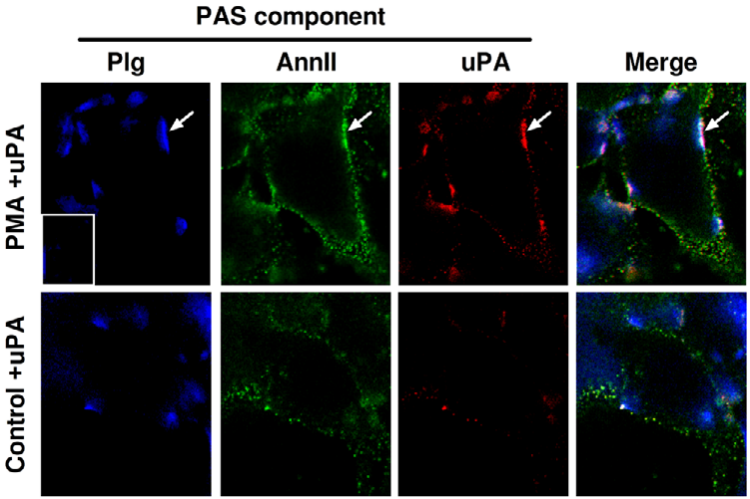
PMA stimulation prior to urokinase plasminogen activator (uPA) incubation mediates increased plasminogen (plg) binding at the MCF-7 cell surface. Confocal microscopy images of control and PMA-stimulated (100 nM, 16 hours) MCF-7 cells both incubated with 50 nM uPA for 10 minutes at room temperature, washed, and probed for annexin II (AnnII), uPA, and Glu-plg binding. Areas of co-localisation of annexin II, uPA, and Glu-plg are shown in white in the merged image. Arrows indicate areas of concentrated expression. Inset shows non-lysine-dependent plg binding (that is, plg binding in the presence of 5 mM tranexamic acid). Glu, glutamic acid; PAS, plasminogen activation system; PMA, 12-O-tetradecanoylphorbol 13-acetate.

To investigate the spatial relationship between cell-surface-bound plg and putative plg receptors, we performed co-localisation experiments using immunofluorescence staining and confocal microscopy. Results for both control and PMA-stimulated MCF-7 cells pre-incubated with exogenous uPA are shown (Figure 4). In the absence of exogenous uPA, regardless of PMA stimulation, very little uPA staining was observed (data not shown).

In MCF-7 cells, annexin II displays punctuate cell-surface and diffuse intracellular staining. After PMA stimulation, there was both a subtle increase and a distinct redistribution of cell-surface annexin II into clustered surface puncta (Figure 4). A similar localisation pattern was observed for exogenous uPA in response to PMA stimulation, reflecting the large increase in uPAR expression compared to control cells (Figure 4). After PMA stimulation, there also appeared to be a visible increase in cell-surface-bound plg, which was subtle and concentrated in specific regions (Figure 4). Plg binding was lysine-dependent given that binding was significantly decreased in the presence of tranexamic acid (Figure 4, inset). Importantly, clear co-localisation of uPA, annexin II, and plg was observed at the surface of these cells after PMA stimulation and exogenous uPA treatment (Figure 4). Very little co-localisation was observed in uPA-treated unstimulated control cells. Because urine-derived uPA was used, these data indirectly show that uPA, annexin II, and plg can co-localise independently of an uPA A-chain/plg interaction after PMA stimulation.

### uPA proteolytic capacity is the critical determinant of plg binding and activation

**Figure 5:**
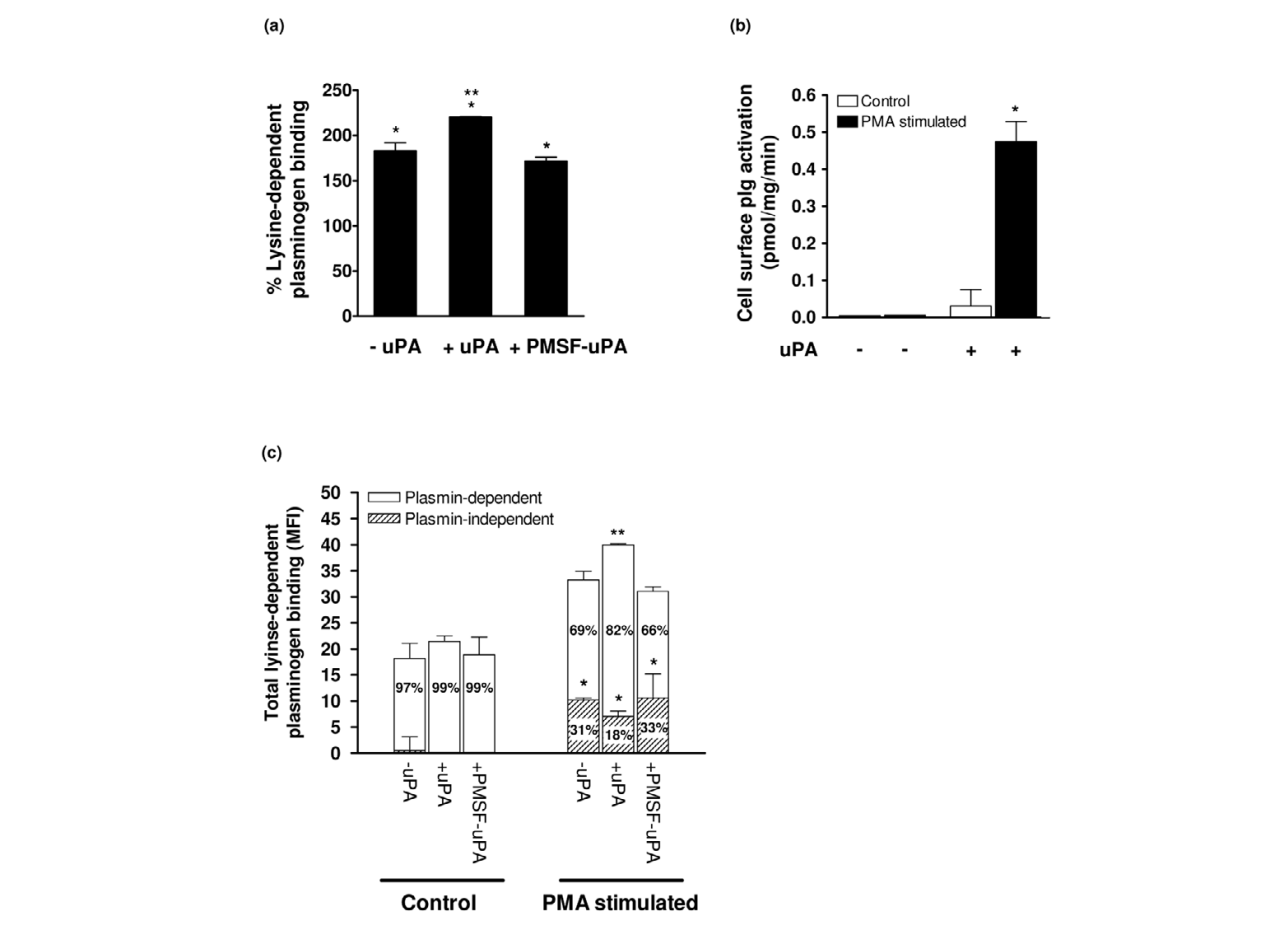
Plasminogen binding and activation on MCF-7 cells. After culture for 16 hours in 5% foetal calf serum/RPMI containing 100 nM PMA (PMA-stimulated) or vehicle alone (control), cells were detached and pre-incubated for 10 minutes at room temperature in the absence or presence of 50 nM active urokinase plasminogen activator (uPA) or PMSF-inactivated uPA (PMSF-uPA), washed, and then analysed for cell-surface plg binding or activation. **(a) **Cell-surface, lysine-dependent plg binding to PMA-stimulated MCF-7 cells is shown as a percentage increase compared to unstimulated MCF-7 cells in the absence of uPA. **(b) **Cell-surface plasmin (pln) generation. Pln activity assays were performed using Spectrozyme PL in the presence of α_2_-antiplasmin to inhibit any solution-phase pln generation. Activity in the presence of aprotinin (pln inhibitor) was also measured and subtracted from all values to determine pln-dependent activity. **(c) **Cell-surface lysine-dependent fluorescein isothiocyanate-plg binding was measured in the presence or absence of aprotinin. Percentages show the proportion of binding due to pln activity at the cell surface (that is, pln-dependent binding calculated as total binding minus binding in the presence of aprotinin; open bars) and the proportion that is independent of pln activity (that is, pln-independent binding calculated as residual binding in the presence of aprotinin; hatched bars), which together constitute total lysine-dependent plg binding. *Significant increase compared to unstimulated control cells not pre-incubated with uPA or PMSF-uPA (*p *< 0.05). **Significant increase compared to PMA-stimulated cells not pre-incubated with uPA or PMSF-uPA. MFI, mean fluorescence intensity; PMA, 12-*O*-tetradecanoylphorbol-13-acetate; PMSF, alpha-toluenesulfonyl fluoride.

Several studies in other cell types have shown that limited proteolysis of the cell surface by pln can reveal cryptic plg-binding sites [21,22,40]. To assess the effect of cell-surface proteolytic activity on plg binding, a number of experiments were performed on MCF-7 cells with modulated active and inactive exogenous uPA levels. No significant difference in lysine-dependent plg binding was observed in control cells pre-incubated with either uPA or PMSF-uPA (data not shown). However, lysine-dependent plg binding on MCF-7 cells after PMA stimulation increased to approximately 183% compared to control cells (Figure 5a). Subsequent addition of exogenous uPA caused a further approximately 37% increase in plg binding, which was not observed with the addition of PMSF-uPA (Figure 5a). Given that the direct, non-active-site-dependent interaction of plg with uPA was precluded through the use of urine-derived uPA, this clearly demonstrates that the additional plg binding observed after uPA treatment of PMA-stimulated cells is entirely dependent on uPA catalytic activity. This effect is functionally important because PMA stimulation alone (which increased plg binding) had no effect on plg activation, whereas uPA treatment of these cells caused a very large (15-fold) increase in cell-surface pln generation (Figure 5b). In addition, the pln generated represents activated cell-surface-bound plg as solution-phase pln was inhibited by the presence of α_2_-antiplasmin. Hence, without uPA catalytic activity at the cell surface, increased plg binding alone is not sufficient for increased plg activation. Nevertheless, increased lysine-dependent plg binding has been clearly linked to increased plg activation in the presence of uPA activity [27].

To further dissect lysine-dependent plg binding mechanisms in our experimental model, experiments were performed in the presence of the pln inhibitor aprotinin to allow a distinction to be made between pln-dependent binding (total binding minus binding in the presence of aprotinin) and pln-independent binding (residual binding in the presence of aprotinin). Plg binding on control (unstimulated) cells, regardless of uPA treatment, was entirely pln-dependent as very little residual plg binding was observed in the presence of aprotinin (Figure 5c). In contrast, PMA stimulation caused an increase in plg binding, which appears to be due to a corresponding increase in pln-independent binding (denoted by the hatched bars in Figure 5c). This pln-independent increase in plg binding may be due to the *de novo *expression of plg-binding moieties after PMA stimulation. Furthermore, amongst the PMA-stimulated cells, the additional and significant increase in plg binding observed after exogenous active uPA treatment was entirely pln-dependent given that there was no corresponding increase in residual plg binding in the presence of aprotinin (Figure 5c). A similar binding relationship was observed on unstimulated MDA-MB-231 cells (which already have a high capacity for plg and uPA binding and activation [27]), where pln-dependent binding accounted for approximately 64% and pln-independent binding for 36% of the total lysine-dependent plg binding. Taken together, these data indicate the importance of increased uPA proteolytic capacity for increased cell-surface plg binding and activation but also suggest a role for pre-formed receptors.

## Discussion

A number of clinical and functional studies have shown that uPA-mediated plg activation is integral to the processes of cancer cell invasion and metastasis [1,2,6]. We have used techniques that maintain cell viability (and hence exclude intracellular plg binding from the analyses) to confirm that the majority of plg binding on breast cancer cells is regulated by pln activity at the cell surface. We show that regardless of the level of plg bound, active uPA must be present at significant levels in order for increased plg activation to occur, and we confirm that a proportion of plg binding to the cell surface is mediated via a region in the endogenous (pro)-uPA A-chain. Furthermore, we demonstrate that annexin II is present at high levels on the surface of MCF-7 cells and co-localises with plg and uPA/uPAR under conditions of increased cell-surface plg binding.

Progression to the metastatic phenotype is clearly associated with deregulation of the uPA system [1,24,41,42], which also appears to be coupled with an increase in pre-formed plg receptors [28]. Plg bound at the cell surface may then be activated to pln by cell-surface or possibly stromal cell-derived [43-46] uPA. The increased uPA expression associated with metastatic cancer increases pln activity, catalysing extracellular matrix degradation and promoting migration [47]. We have previously shown that metastatic breast cancer cells (for example, MDA-MB-231) have increased uPA at the cell surface and have the potential to bind and activate relatively large amounts of plg compared to nonmetastatic cells (for example, MCF-7 and T-47D) [27]. In a clinical setting, strong immunohistochemical staining of uPA has been significantly correlated to more invasive tumours [48,49] and uPA constitutes a strong, statistically significant independent prognostic marker for shorter disease-free survival [50-52]. Together, these results suggest that uPA has a key role in regulating metastatic potential by modulating cell-surface plg binding and activating capacity and hence proteolytic activity.

Pro-uPA, though termed an 'inactive' precursor, has been shown to possess some minimal intrinsic plg activating capability [53]. In a proposed model based on studies of monocytic and colon cancer cell lines, cells with significant amounts of pro-uPA at the cell surface are able to activate bound plg, the pln formed then exposes lysine residues through limited proteolysis of nearby proteins and in addition activates pro-uPA to the more efficient uPA [40,54]. The newly bound plg is then activated, creating a positive feedback activation loop between (pro)-uPA and plg/pln which has the potential to generate large amounts of pln at the cell surface. Our results provide further support for this mechanism of action on breast cancer cells. MCF-7 cells possess low endogenous cell-surface (pro)-uPA levels and bind small amounts of cell-surface plg [27]. As a result, they possess comparatively little plg activating ability and display only minimal invasive activity [27]. By enhancing uPAR levels and using urine-derived uPA to preclude direct lysine-dependent binding, we clearly confirm that plg binding is largely dependent on uPA activity and subsequent pln activity and that pln does play a key role in the generation of new plg-binding sites at the cell surface (Figure 5).

The ability of pln to generate its own plg-binding sites in a positive feedback loop (independent of *de novo *synthesis) has been demonstrated on neutrophils and monocytic and colon cancer cell lines by pretreatment of cells with pln [21-23]. Additionally, carboxypeptidase treatment to remove C-terminal lysine residues from proteins has been shown to cause marked decreases in plg binding in a number of cell types [20,22,55]. In our experience, however, pre-treatment with high concentrations of pln at 37°C devastates cell viability *in vitro*. Moreover, we have previously shown that non-viable cell populations bind approximately 100 times more plg, masking changes in cell-surface binding on viable cells [27]. Thus, the use of ^125^I-plg (which cannot distinguish between intracellular and cell-surface binding) to measure cell-surface plg binding is unsuitable because considerable artifactual contributions of intracellular plg binding cannot be excluded. In contrast, the use of dual-colour flow cytometry is far superior because it allows clear distinction of cell-surface from intracellular plg binding by exclusion of non-viable cells. Furthermore, the use of aprotinin in this study to quantify the contribution of pln activity to plg binding removes the inherent complicating effect of pln treatment on cell viability. These modifications therefore represent significant improvements to previous works and allow careful characterisation of cell-surface events.

A number of proteins, including actin, AIIt (consisting of two molecules of annexin II [p36 subunit] and two molecules of S100A10 [p11 subunit]), cytokeratin 8, and α-enolase, have been shown to both bind and enhance the activation of plg to pln on a number of cell types [1]. The nature of cell-surface plg binding and activation (that is, low-affinity, lysine-dependent) suggests a general requirement for exposed lysine residues at the cell surface rather than the presence of a specific receptor or suite of receptors to promote plg binding and activation. We did not observe significant expression of actin or cytokeratin 8/18 at the MCF-7 cell surface under any condition. However, MCF-7 cells have relatively high endogenous levels of annexin II at the cell surface (Figures 2 and 4). Annexin II has been shown to exist in monomeric, heterodimeric, and heterotetrameric forms [56] and associates with the cell surface in a calcium-dependent manner [57]. Initially, it was reported that annexin II on endothelial cells bound tPA, plg, and pln [58], suggesting that although native annexin II does not contain a C-terminal lysine residue, cleavage of annexin II at the Lys_307 _residue by pln-like serine protease activity may expose a new plg/pln binding site. Further studies with purified proteins have demonstrated enhanced tPA-mediated plg activation in the presence of annexin II monomer [59], again suggesting that plg can bind to annexin II directly. However, proteolytically processed annexin II has not been shown to exist either *in vitro *or *in vivo *and denatured protein has been demonstrated to stimulate pln formation non-specifically [60].

Surface plasmon resonance studies have demonstrated that only AIIt or isolated p11 subunit (S100A10) is able to bind tPA, plg, and pln with moderate affinity, whereas annexin II subunit is able to bind only pln [60,61]. P11 expression is regulated by annexin II via a post-translational mechanism in various epithelial cell lines and it has been suggested that plg binding to AIIt occurs primarily via the Cterminal lysine on the p11 subunit [61-63]. We observed pln-independent upregulation of the p11 subunit after PMA stimulation (Figure 2), associated with an increase in plg binding. Our confocal microscopy data showed a distinct redistribution of annexin II, and a proportion co-localised with plg and uPA/uPAR in PMA-stimulated cells (Figure 3). Because p11 expression is increased by PMA stimulation and has been shown to co-localise with uPAR [60], it is possible that the pln-independent increase in plg binding may be partially mediated by the increase in p11. However, the overall amount of p11 was small, even after PMA stimulation, and we could not detect p11 by confocal microscopy. Therefore, p11 may account for only a very small proportion of plg binding in these cells. Thus, the presence of both protease-dependent and -independent plg receptors on modified MCF-7 and MDA-MB-231 cells serves to indicate that both types of receptor contribute to the total lysine-dependent plg-binding capacity on invasive cells. This reflects the increased suite of both uPA-localised and non-co-localised plg-binding sites on more malignant cells such as MDA-MB-231 [28], which the PMA-stimulated and uPA-treated MCF-7 cells mimicked.

The invasive capacity of breast cancer cells may be linked to the presence of significant levels of active uPA, which in concert with even a moderate amount of bound plg can generate large amounts of pln at the cell surface through a positive feedback mechanism. Mounting evidence points to uPA as an integral facilitator of breast cancer progression. Recent studies have indicated that uPA deficiency in a breast cancer mouse model significantly reduces lung and lymph node metastases [64], and recent findings by our group indicate that targeting of uPA (of either tumour or stromal cell origin) with a radiolabelled ligand significantly retards the growth of MDA-MB-231 xenografts in nude mice [65]. These studies and others indicate the importance of targeting uPA in anti-invasive tumour strategies.

## Conclusion

This study characterises plg binding and activation at the breast cancer cell surface using techniques that exclude artifacts derived from intracellular proteins, contributions from non-viable cells, and the C-terminal lysine from the uPA A-chain. We show for the first time that several mechanisms regulate breast cancer cell plg binding, including direct binding of plg to uPA/pro-uPA, expression of various plg binding proteins, and pln-mediated generation of plg-binding sites through limited proteolysis of cell-surface proteins. Critically, cells with increased uPA and a moderate increase in plg binding displayed dramatic increases in pln activity. This effect was not observed in MCF-7 cells with moderate plg binding alone but was observed in MDA-MB-231 cells, which have naturally high levels of uPA, pln generation, and invasive capacity. Our data thus support the model that increased uPA expression at the cell surface substantially increases pln generation, and therefore invasive potential, via a positive feedback mechanism. These results also strengthen the evidence implicating uPA as a strong therapeutic target for the treatment of metastatic breast cancer.

## Abbreviations

AIIt = annexin II heterotetramer; Cy = cyanine; FCS = foetal calf serum; FITC = fluorescein isothiocyanate; Glu = glutamic acid; IgG = immunoglobulin G; *K*_d _= dissociation constant; mAb = monoclonal antibody; MBB = modified (no phenol red) binding buffer; MFI = mean fluorescence intensity; pAb = polyclonal antibody; PAI-1 = plasminogen activator inhibitor type-1; PAI-2 = plasminogen activator inhibitor type-2; PAS = plasminogen activation system; PBS = phosphate-buffered saline; PI = propidium iodide; plg = plasminogen; pln = plasmin; PMA = 12-*O*-tetradecanoylphorbol-13-acetate; PMSF = alpha-toluenesulfonyl fluoride; PMSFuPA = alpha-toluenesulfonyl fluorideinactivated urokinase plasminogen activator; TIU = trypsin inhibitor units; tPA = tissue-type plasminogen activator; uPA = urokinase plasminogen activator; uPAR = urokinase plasminogen activator receptor.

## Competing interests

The authors declare that they have no competing interests.

## Authors' contributions

GES contributed to the study concept and design, data acquisition, data analysis/interpretation, and manuscript drafting and editing. DNS contributed to data analysis/interpretation and manuscript drafting and editing. MR contributed to the study concept and design, data analysis/interpretation, and manuscript drafting and editing. All authors read and approved the final manuscript.
